# Cauda equina syndrome caused by *filum terminale* lipoma: magnetic resonance imaging features and surgical treatment

**DOI:** 10.1590/S1679-45082017AI3882

**Published:** 2017

**Authors:** Benedito Jamilson Araújo Pereira, Ulysses Caus Batista, Fúlvio Nicolau Bechelli, Carlos Alberto Afonso Ribeiro, Carlos Vanderlei Medeiros de Holanda, Paulo Eduardo Carvalho Galvão

**Affiliations:** 1 Hospital Beneficência Portuguesa, São Paulo, SP, Brazil.; 2 Universidade Estadual de Campinas, Campinas, SP, Brazil.

**Figure 1:**
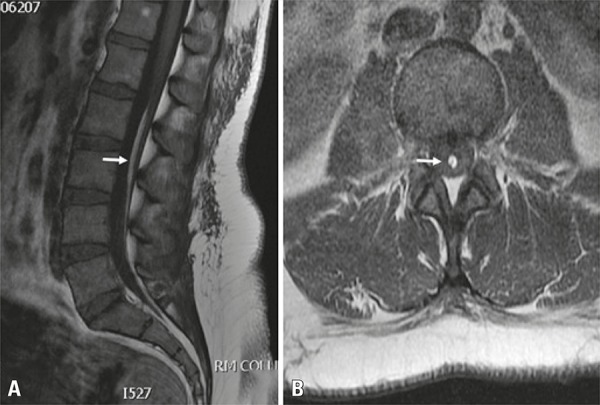
Pre-operative images. T1-weighted magnetic resonance images showing a lipoma of the *filum terminale* (hyperintense sign within the *filum terminale*, white arrow) in sagittal (A) and axial (B) planes

**Figure 2:**
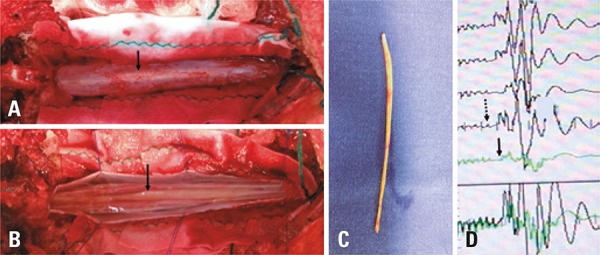
Operative findings surgical features: exposure of the dural sac (A), observe the lipoma of the *filum terminale* (arrow) and after dural sac open (B). Lipoma *filum terminale* (C), neurophysiological monitoring (D): tracings demonstrating amplitude of the wave and the number of stages before (continuous arrow) and after (dashed arrow). An increase was observed in amplitude of wave number and phase after resection of the lipoma of the *filum terminale*

A 36-year-old woman with a sudden history of low back pain radiating into the right lower extremity. The physical examination revealed paraparesis, saddle anesthesia and urinary incontinence. Magnetic resonance imaging revealed lipoma of the *filum terminale* (hyperintense sign within the *filum terminale*) (Figure 1). The patient underwent surgery without delay with intraoperative neurophysiological monitoring (Figure 2), and she had a full recovery of all neurological *deficits*.

Lipoma of the *filum terminale* is one of the commonest causes of occult spinal dysraphism.^1^ However, only few articles associate lipoma of the *filum terminale* and neurological *deficit*.^2^
